# Association of periodontitis with cardiometabolic and haemostatic parameters

**DOI:** 10.1007/s00784-024-05893-y

**Published:** 2024-08-30

**Authors:** Hester Groenewegen, Jaime F. Borjas-Howard, Karina Meijer, Ton Lisman, Arjan Vissink, Fred K. L. Spijkervet, Willem Nesse, Vladimir Y. I. G. V. Tichelaar

**Affiliations:** 1 Department of Oral and Maxillofacial Surgery, University of Groningen and University Medical Center Groningen, P.O. Box 30.001, Groningen, 9700 RB The Netherlands; 2 Department of Haematology, University of Groningen and University Medical Center Groningen, P.O. Box 30.001, Groningen, 9700 RB The Netherlands; 3 Department of Surgical Research Laboratory, University of Groningen and University Medical Center Groningen, P.O. Box 30.001, Groningen, 9700 RB The Netherlands; 4 Department of Oral and Maxillofacial Surgery, Wilhelmina Hospital Assen, Postbus 30001, Assen, 9400 RA The Netherlands

**Keywords:** Cardiovascular diseases, Periodontitis, Tooth extraction, Inflammation, Venous thromboembolism

## Abstract

**Objective:**

To investigate the association between periodontitis and cardiometabolic and haemostatic parameters.

**Materials and methods:**

Between 2014 and 2019, 54 individuals needing full mouth extraction, and 50 control individuals, were recruited for a combined cross-sectional (individuals versus controls) and longitudinal (individuals before and after extraction) study. Periodontitis severity was measured using the periodontal inflamed surface area (PISA). Blood was drawn to measure the haemostatic (Factor VIII, von Willebrand factor [VWF], endogenous thrombin potential, d-dimer, clot lysis time) and cardiovascular risk (C-reactive protein [CRP], lipid profile) parameters, prior to and 12 weeks post-extraction. The results were analysed group-wise.

**Results:**

The mean VWF and CRP levels were higher and the high-density lipoprotein levels were lower in the individuals prior to extraction compared to the controls. The VWF was significantly correlated with the PISA (a 21% unit increase in VWF per 1000 mm^2^ increase in PISA, 95%CI: 6–36%, *p* = 0.01). The other analyses were comparable between the individuals and controls, and did not change in the individuals after the extraction.

**Conclusion:**

VWF levels are associated with periodontitis severity; they do not improve after full-mouth extraction. Severe periodontitis in control individuals does not induce substantial changes in their haemostatic or inflammatory systems.

**Clinical relevance:**

Treatment of periodontitis has been shown to improve the cardiometabolic blood profile of patients with established cardiometabolic disease. However, whether periodontitis treatment improves cardiometabolic and haemostatic profiles in people without cardiometabolic disease is uncertain.

**Supplementary Information:**

The online version contains supplementary material available at 10.1007/s00784-024-05893-y.

## Introduction

Ongoing research has established that a wide range of inflammatory processes, such as acute/chronic infections or autoimmune diseases, are risk factors for both arterial and venous thrombotic events [1, 2]. Periodontitis is a chronic inflammatory process of the gums and deeper periodontal structures that results in destruction of the periodontal ligament and alveolar bone, with gingival recession and periodontal pocket formation [3].

Periodontitis has been shown to have systemic effects as well [4, 5], which likely caused by, haematogenous dissemination of bacterial translocation of oral microbiota or a spillover of inflammatory mediators from periodontal tissues to into the blood stream from the inflamed periodontium. A causal association between periodontitis and arterial as well as venous cardiovascular disease seems plausible and, indeed, observational studies established an association with arterial cardiovascular disease [6–8].

However, observational studies are prone to residual confounding. To assert causality more reliably, periodontal intervention studies are needed to assess whether they lead to a lower risk of cardiovascular disease. Due to the ethical problems in withholding periodontal treatment long enough to observe an effect on incident cardiovascular disease, studies have focused on the effect of an intervention on the underlying cardiometabolic blood parameters, using it as a surrogate for cardiovascular disease. Teeuw et al. (2014), published their results of a meta-analysis assessing an association between periodontitis treatment and changes in cardiometabolic biomarkers as a surrogate for cardiovascular disease [9]. Overall, the meta-analysis showed that an improvement in biomarker profile was observed in individual in the intervention arms, the primary result being a decline in C-reactive protein (CRP) levels. However, it became apparent from the subgroup analysis that these beneficial effects were limited to individuals who were already suffering from established cardiovascular disease or had diabetes. The effect was not seen in individuals without established disease. Hence, whilst periodontitis treatment might benefit individuals with established cardiometabolic disease or with hypertension and no established cardiovascular diseases, the potential benefit of periodontitis treatment in people without cardiometabolic disease remains unclear [10, 11].

Moreover, a paucity of data exists on the association between periodontitis and venous thromboembolism (VTE), for which there is also a well-established association with inflammatory disease processes [12]. Two trials and one one-armed intervention study assessed the effect of periodontal interventions on selected haemostatic parameters (von Willebrand factor [VWF], tissue plasminogen activator, plasminogen activator inhibitor 1 and fibrinogen) and found no association [13–15]. More recent research has established other haemostatic measures with stronger causal links to VTE, namely d-dimer, the endogenous thrombin potential and clot lysis time [16–18]. Therefore, it is valuable to reassess the association between periodontitis and VTE using a broader range of haemostatic parameters and a large intervention such as full mouth dental extraction in individuals with periodontal disease. The periodontal inflamed surface area (PISA) is used to reflect the surface area of bleeding pocket epithelium in square millimetres. It is assumed that PISA quantifies the inflammatory burden posed by periodontitis [19].

Hence, the aim of the current study was to investigate the association between periodontitis and both cardiometabolic and haemostatic parameters in individuals free of established cardiometabolic disease and VTE, in a full mouth dental extraction setting. Individuals for whom a full mouth dental extraction is indicated usually have higher levels of periodontitis than the average population [20, 21] We presumed that a full mouth dental extraction entails removing all the periodontitis tissue, and thus any systemic inflammatory burden resulting from the periodontitis will also be completely removed.

## Methods

### Design

This study entailed both a cross-sectional and longitudinal experimental design. In the former, we compared the individuals referred for full mouth dental extraction at baseline with control individuals from the community. Regarding the latter, we compared the before and after extraction outcomes. The indication for full mouth extraction was set by the treating dentist. The research question was developed following the PICO method:

- P: Individuals needing a full mouth dental extraction due to periodontitis and extensive decayed dentition.
- I: Full mouth dental extraction.
- C: 1: Individuals matched for age and sex, but not having a desolate oral heath.
- 2: Initially included individuals after the full mouth dental extraction.
- O: PISA, levels of cardiometabolic and haemostatic parameters.

### Participants

Individuals aged 18 years or above referred by their dentist for removal of all their remaining teeth (full mouth dental extraction) due to periodontitis and extensive decay dentition to the outpatient department of oral and maxillofacial surgery in the University Medical Center Groningen and the Wilhelmina hospital Assen were screened for individual eligibility. Participants were excluded if they were unable to understand spoken/written Dutch and/or English, had documented liver dysfunction, were taking antithrombotic medication or non-steroidal anti-inflammatory drugs, suffered from chronic autoimmune disease and/or had a history of radiotherapy of the head/neck. The included patients had most cases severely decayed dentition and severe anxiety for oral examination/intervention.

Control individuals were recruited by poster advertisements in supermarkets and around the hospital for oral health status comparisons with the participating individuals. The control individuals had to be within the age range of the recruited individuals, but should not have a desolate dentition. Therefore, the recruitment of volunteers began after the 20 individuals who had been referred for a full mouth dental extraction were already included in the intervention arm. The exclusion criteria for the control individuals were the same as for the subject group.

### Measurements

After signing the informed consent, all the participants were asked to complete a standardized questionnaire, and were subjected to a venepuncture and an assessment of their periodontal status.

#### Venepuncture

Blood was drawn from the antecubital fossa: 18 ml blood was drawn into citrate vials to measure haemostatic parameters; 9 ml of blood was drawn into lithium-heparin for routine biochemistry assessments, including serum insulin and lipid profile; 4.5 ml blood was drawn into EDTA vials for a full blood count; and 4.5 ml blood was drawn into fluoride vials to measure fasting blood glucose. For individuals undergoing tooth extraction we mandated citrate blood samples to be drawn maximal 6 weeks before the intervention during the pre-operative screening as we were concerned that pre-procedural stress/anxiety may influence haemostatic parameters- a citrate blood sample drawn day prior to the procedure was deemed acceptable. To investigate the effects of the extraction on haemostatic parameters, a second venepuncture was performed at least 12 weeks after the procedure. This minimum time frame was assumed to be sufficient for wound healing.

The citrate blood samples were centrifuged twice (2,000 g and subsequently 10,000 g) for 10 min. The samples were stored in a -80 °C freezer within 4 h of the venepuncture to be analysed batchwise.

For participants in the control group, blood was drawn the day of periodontal examination, prior to assessing the periodontal status because any pain due to this assessment could influence haemostatic parameters.

#### Laboratory outcomes

Haemostatic parameters consisted of prothrombin fragment 1 + 2, factor VIII, von Willebrand factor, endogenous thrombin potential (in the presence and absence of exogenously added soluble thrombomodulin) and its subcomponents (peak thrombin, velocity index, lag time), D-dimer and clot lysis time. The assay details can be found in the appendix.

The predefined laboratory measurements regarding cardiovascular risk biomarkers were high sensitivity CRP, total cholesterol, low-density lipoprotein, high-density lipoprotein (HDL-c ), and HOMA 1 insulin resistance model consisting of a calculation combining fasting plasma glucose and serum insuline levels, which was updated to the HOMA 2 model post-hoc [22].

#### Periodontal measurements

The periodontal examination of the individuals who were scheduled to have a full mouth dental extraction was done under general anaesthesia (most of them were anxious about routine dental care) just before the full mouth clearance. All the periodontal examinations were executed by the same experienced dental hygienist (HG). Probing pocket depth (PPD), gingival recession, bleeding on probing and clinical attachment level (CAL) were measured in millimetres (Williams probe 14 W, Hu-Friedy Mfg. Co., LLC, UK), rounded to the nearest whole millimetre. Bleeding on probing was recorded as either present or absent 30 s after probing. The number of missing teeth was recorded. All these measurements were entered into a spreadsheet to calculate the periodontal inflamed surface area (PISA); this quantifies the surface area of inflamed periodontal tissue in square millimetres and is a quantitative measure of the inflammatory burden posed by periodontitis [19]. The presence of periodontitis was also defined according to the CDC-AAP case definition of periodontitis surveillance for epidemiologic studies. Mild periodontitis was recorded for cases with ≥ 2 interproximal sites with a CAL ≥ 3 mm and ≥ 2 interproximal sites with a PPD ≥ 4 mm (not on the same tooth) or 1 site with a PPD ≥ 5 mm. The participants were classified as having moderate periodontitis in the presence of ≥ 2 interproximal sites with a CAL ≥ 4 mm (not on the same tooth) or ≥ 2 interproximal sites with a PPD ≥ 5 mm, also not on the same tooth. Severe periodontitis was recorded if the participants had ≥ 2 interproximal sites with clinical attachment loss, a CAL ≥ 6 mm, not on the same tooth, and ≥ 1 interproximal site with a PPD ≥ 5 mm [23–25].

#### Questionnaire

Data were collected about exposure to VTE risk factors (i.e., surgery, immobilization, long-haul travel, use of hormones) in the preceding 3 months, educational status, smoking status and whether there were any relevant comorbidities (including previous VTE) present. The data about the individuals’ heights and weights measured at the anaesthesiology outpatient clinic were extracted from the records. The control individuals’ heights and body weights were self-reported.

### Statistical analysis

The study was powered to detect a 10% difference in endogenous thrombin potential 12 weeks after full mouth extraction, assuming a mean of 1050 nM IIa *min, SD 200 nM IIa *min with 80% power at alpha 0.05. To detect this difference, we needed 50 individuals. Individuals lost to follow-up after the extraction were supplemented with extra recruits until there were 50 individuals available for the longitudinal analyses. The controls were recruited to achieve a 1:1 ratio without a formal sample size calculation.

The normally distributed laboratory measurements were described as means with standard deviations and the not normally distributed ones were described as medians and interquartile ranges.

The between group (cross-sectional) analysis consisted of a comparison between the full mouth extraction individuals and the controls. Either the students t-test or the Wilcoxon rank sum test was used accordingly. A linear regression model was built for all the participants, with the laboratory measurement of interest as the dependent variable and PISA, modelled continuously in 1,000 mm^2^ increments, as the main independent variable of interest. The coefficients were adjusted for age, sex, educational status, smoking status (categorized as current, former and never) and BMI, modelled as a continuous variable.

In the intervention (longitudinal) analysis, paired differences were tested using a paired t-test or Wilcoxon signed-rank test. Regarding the regression analysis, the change in laboratory values was calculated by subtracting the value of the first value from the first from the second measurement (T_2_-T_1_) and defined this as the dependent variable, with PISA modelled as the independent variable. These coefficient values were not adjusted– nor was the baseline value– as suggested by Glymour et al. ( 2005) [26].

The assumptions of the linear models were checked by assessing residual normality. If the assumptions were violated, the models were rerun by first transforming the dependent variable, then by transforming the PISA, and subsequently by transforming both.

Sensitivity analyses were carried out by removing outlier values with specific characteristics, these were done post-hoc. First, the general outlier values were excluded, defined by examining the scatter plots and by examining the Cook’s distance in the linear regression models. Second, participants reporting antibiotic use at any time before their first measurement were excluded. Third, the participants with pathological outlying CRP values (defined as > 10 mg/L) were excluded. The underlying assumption here was that these values may have represented participants with an underlying (subclinical) infection, or recovering from such an infection. Moreover, we excluded the participants who had recently been exposed to a VTE risk factor, as this may have distorted haemostatic parameter results. Additionally, we explored the impact of potential measurement error in the control group’s self-reported BMI by a correction formula suggested by Dutton et al. (2014) [27] and refitted the cross-sectional regression models with these imputed BMI values. Additionally, because we found a linear relationship between HDL-C levels and PISA extracted, we tested whether this linear relationship was consistent for relatively low baseline levels of HDL-c, as a meta-analysis of HDL-C levels suggest a J/U shaped relationship between HDL-c levels and all-cause/cardiovascular mortality. In this meta-analysis, the lowest mortality was found for HDL-c levels around 1,5 − 1,7mmol/l. We thus reran models considering only participants with baseline values below this [28]. Finally, at peer review, a suggestion was made to model alternative metrics of smoking. We initially chose categorical smoking status (never/former/current smoking) as this had the lowest frequency of missing data. A brief literature search suggested that smoking intensity might be better predictor of cardiometabolic risk status [29]. Missing data with regards to smoking intensity was enriched by consulting referral letters and current smoking intensity was modelled as current amount of cigarettes smoked daily.

## Results

**Fig. 1:**
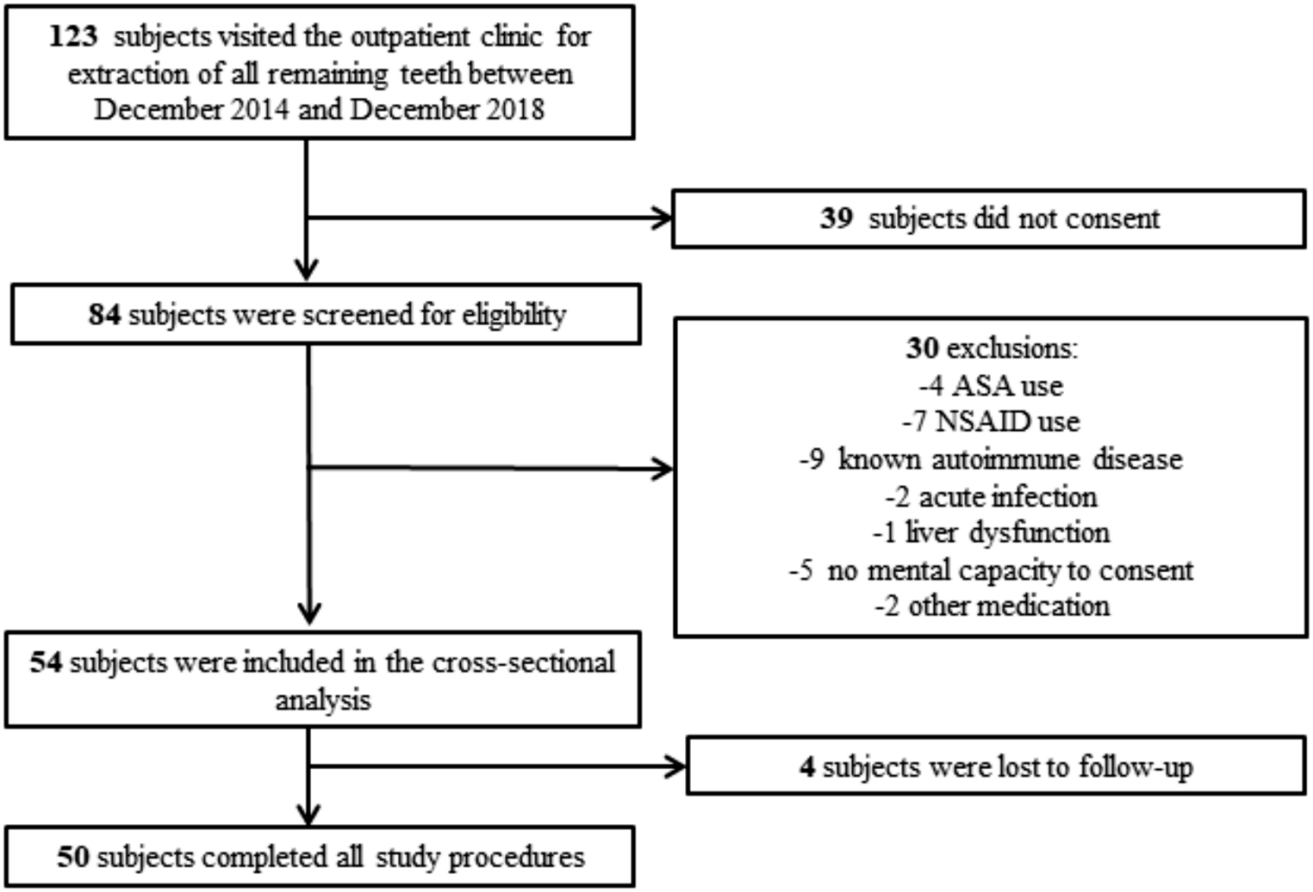
Flowchart depicting recruitment of individuals undergoing full mouth extraction

One hundred and twenty-two individuals visited the outpatient clinic for full mouth dental extraction between December 2014 and December 2018. Eighty-three individuals were screened for eligibility and 54 were recruited. We recruited an additional 4 individuals because 4 were lost to follow-up after the teeth removal: we tried to contact these individuals with repeated phone calls and correspondence to their last known home addresses, but to no avail. The latter four individuals were thus included in the cross-sectional analysis. Fifty control individuals were recruited in the same way as described above. Therefore, for a total of 104 individuals, data were available for the cross-sectional analysis and 50 for the longitudinal analysis. A flowchart showing the individuals selection in the intervention group is depicted in Fig. 1.

Table 1 shows the baseline characteristics within the two study arms. The arms were comparable regarding age and proportion of men participating. As expected, the PISA was higher in the intervention group compared to the control group. Also the individuals with severe periodontitis was much higher in the intervention group (98%), compared to 22% with severe periodontitis in the control group. In the intervention group there were no individuals with mild periodontitis and 2% with moderate periodontitis. In the controls 16% was having mild periodontitis and 62% moderate periodontitis. An overlap occurred mainly because 3 individuals in the intervention group only (< 10) teeth left for removal. The intervention individuals had a slightly higher BMI, had on average a lower educational status and were more frequently smokers than the control individuals.

**Table 1:** Baseline characteristics

	Full mouth dental extraction (*n* = 54)	controls individuals (*n* = 50)
Mean age (SD)	45 (10,5)	48 (12,6)
Male sex n (%)	29 (54%)	24 (48%)
BMI (SD)	26,7 (6,4)	24,8 (3,1)
n missing	0	6
Median PISA in mm (IQR)	2004 (1082–2613)	853 (463–1088)
Periodontitis classification (%)		
Mild periodontitis	-	8(16%)
Moderate Priodontitis	1(2%)	31(62%)
Severe Periodontitis	53(98%)	11(22%)
Median number of elements (IQR)	22 (15–25)	27 (26–28)
History of VTE (%)	3 (6%)	1 (2%)
Other VTE risk factors (%)	9 (17%)	10 (20%)
Education level (%)		
secondary only	15 (28%)	4 (8%)
vocational education	35 (65%)	24 (48%)
college or higher	3 (6%)	21 (42%)
N Missing	1 (2%)	1 (2%)
Smoking		
never	6 (11%)	27 (54%)
former	5 (9%)	12 (24%)
current	43 (80%)	10 (20%)
Median number of cigarettes in current smokers group(IQR)	15(10–20)	10 (10–15)
N Missing		1
Hypercholesterolemia (%)	6 (11%)	5 (8%)
Hypertension (%)	4 (7%)	3 (5%)
Diabetes (%)	2 (4%)	0 (0%)
Use of antibiotics (%)	5 (9%)	2 (4%)

BMI: body mass index; PISA: Periodontal inflamed surface area; VTE: venous thromboembolism

Table 2 shows the laboratory results of the cross-sectional analysis. The mean VWF and CRP levels were higher in the full mouth dental extraction individuals compared to the control group, while their HDL levels were lower.

**Table 2:** Cross-sectional laboratory parameter levels

Haemostatic parameters	Full mouth dental extraction (*n* = 54)	control individuals (*n* = 50)	test *p*
Mean Factor VIII % (SD)	119 (33)	110 (31)	0,21
Mean VWF % (SD)	143 (62)	108 (42)	< 0.001
Mean ETP in nM IIa*min (SD)	1131 (223)	1133 (168)	0,95
Median ETP TM in nM IIa*min (IQR)	344 (174–500)	417 (332–592)	< 0.01**
Median d-dimer in ng/mL (IQR)	252 (162–374)	205 (112–400)	0.19*
Median CLT in minutes (IQR)	69 (61–76)	66 (60–72)	0.29**
**Inflammatory/metabolic parameters**			
Median C-reactive protein (IQR)	2,2 (1,1–4,1)	1,0 (0.4–2.2)	0,01*
n missing	3		
Mean TC in mmol/L (SD)	5,2 (1,1)	5,2 (1,1)	0,76
n missing	2		
Mean LDL in mmol/L (SD)	3,6 (1,0)	3,5 (1,0.1)	0.62
Mean HDL in mmol/L (SD)	1.3 (0.4)	1.6 (0.6)	0.01
	1		
Median HOMA2 IR index (IQR)	0.92 (0.69–1.38)	0.9 (0.62–1.23)	0.82**

* Wilcoxon signed rank test; ** t-test after log transformation; ETP: endogenous thrombin potential; TM: thrombomodulin; CLT: clot lysis time; CRP: c-reactive protein; TC: total cholesterol; LDL: low density lipoprotein; HDL: high density lipoprotein; IQR: interquartile range

Table 3 shows the multivariable regression results of the cross-sectional analysis. The scatter plots visually depicting the relationship between the PISA and dependent variables are shown in the appendix. After adjustment for covariates, there was strong evidence for an association between PISA and vWF, but the associations between periodontitis one the one hand with CRP and HDL-c levels seen in the groupwise analysis did not persist after adjusting for confounders.

**Table 3:** Cross-sectional regression analyses results

Laboratory marker	Coefficient* 1000 mm^2^ PISA increment (95% CI)	*p*
Cross-sectional analysis		
Factor VIII	1 (-7 to 10)	0.73
Von Willebrand factor	22 (7 to 37)	0.01
ETP	36 (-13 to 84)	0.14
ETP TM	21 (-23 to 76)	0.43
Dimer (logarithm)	-0.0 (-0.3 to 0.3)	0.99
CLT	-2 (-6 to 2)	0.36
CRP (logarithm)	-0.18 (-0.47 to 0.12)	0.18
Total cholesterol	-0.2 (-0.5 to 0.1)	0.18
LDL	-0.2 (-0.5 to 0.1)	0.10
HDL	0.01 (-0.11 to 0.14)	0.84
HOMA2 IR (logarithm)	0.04 (-0.08 to 0.17)	0.52

* adjusted for age, sex, BMI, smoking status and educational status; ETP: endogenous thrombin potential; TM: thrombomodulin; CLT: clot lysis time; CRP: c-reactive protein; TC: total cholesterol; LDL: low density lipoprotein; HDL: high density lipoprotein

Table 4 describes the intervention individuals’ laboratory values before and after full mouth dental extraction. None of the paired t-tests showed an improvement in the studied parameters before and after tooth extraction. The regression analyses incorporating PISA and changes in laboratory outcomes showed evidence for an elevation of HDL-C levels after full mouth extraction (*p* = 0,02).

**Table 4:** Longitudinal analysis of the full mouth dental extraction individuals (*n* = 50)

Haemostatic parameters	Before	After	Change	95% CI	*p*	Regression coefficient /1000 mm^2^ (95% CI)
Mean Factor VIII, % (SD)	119 (34)	118 (37)	0 (26)	-8 to 8	0.92	1 (-8 to 10)
Mean VWF, % (SD)	143 (58)	141 (54)	-2 (52)	-17 to 12	0.74	5 (-13 to 23)
Mean ETP, nM IIa*min (SD)	1131 (223)	1081 (247)	-48 (240)	-116 to 20	0.16	-32 (-117 to 53)
Median ETP TM, nM IIa*min (IQR)	343 (174 to 500)	300 (211 to 527)	-2 (-81 to 107)	–	0.75*	-11 (-119 to 97)
Median D-dimer, ng/ml (IQR)	252 (162 to 374)	273 (154 to 452)	2 (-79 to 76)	-42 to 43	0.99	-27 (-80 to 26)
Median CLT, min (IQR)	69 (61 to 76)	66 (59 to 77)	-3 (-7 to 4)	–	0.14*	0 (-4 to 4)
**Inflammatory/metabolic parameters**						
median CRP (IQR)	2,1 (0,9 to 3,8)	2,0 (0,9 to 4,3)	-0,1 (-0,7 to 1,0)	–	0.91*	1.0 (-0.8 to 2.7)
n missing	3		3			
mean TC in mmol/L (SD)	5,1 (1,0)	5,2 (1,0)	0 (0,7)	-0,2 to 0,2	0,73	0.16 (-0.08 to 0.40)
n missing	1	1	2			
Mean LDL in mmol/L (SD)	3,6 (1,0)	3,6 (1,0)	-0,1 (0,60)	-0,2 to 0,2	1	0.18 (-0.04 to 0.40)
Mean HDL in mmol/L (SD)	1,3 (0,4)	1,3 (0,3)	0 (0,20)	-0,1 to 0,1	0,83	0.08 (0.01 to 0.14)
HOMA2 IR (IQR)	0.92 (0.69 to 1.38)	0.80 (0.59 to 1.36)	-0.04 (-0.31 to 0.18)	–	0.24	-0.04 (0.25 to 0.18)
N missing***	6	5	7			

* Wilcoxon signed rank test; ** t-test after log transformation; *** includes missing for values that were incalculable vWF: von Willebrand factor; ETP: endogenous thrombin potential; TM: thrombomodulin; CLT: clot lysis time; CRP: c-reactive protein; TC: total cholesterol; LDL: low density lipoprotein; HDL: high density lipoprotein

### Sensitivity analyses

The identification and removal of outliers, the removal of individuals exposed to antibiotics or VTE risk factors, and the correction of self-reported BMIs, did not change the overall results (see section on statistical analyses). Analyses for sex as an interaction variable and did not find any positive results. Limiting the longitudinal analysis to incrementally lower values of baseline HDL-c led to decreasing coefficients in relationship with PISA. Modelling of smoking intensity as opposed to smoking status did not change the results of the cross-sectional analysis. The results of the sensitivity analyses are shown in the appendix.

## Discussion

In the current study we hypothesized that periodontitis severity would be associated with a disturbed state of haemostatic and cardiometabolic biomarkers and, thus, removing the inflammatory burden of periodontitis through a full mouth dental extraction would improve these parameters. Overall, we found limited evidence for this: although we found a solitary association between VWF levels and the amount of inflamed periodontal tissue in the cross-sectional analyses, the VWF levels did not improve after a full mouth dental extraction. This may suggest residual confounding. We found an association between removed inflammatory burden and increase in HDL-c levels. However, given the recently discovered non-linear relationship between HDL-c levels and all-cause/cardiovascular mortality, we amended this analysis, showing decreasing benefits in patients with low baseline HDL-c levels, which were also not significant.

Our study has several conceptual strengths. It complements the data available at the time we designed the study, which showed a limited association between periodontitis and biomarkers, by studying the effects of a more aggressive intervention: a total tooth extraction. We reasoned that, if an aggressive intervention does not produce a significant result, it can be questioned whether less aggressive routine periodontal interventions will be beneficial for hemostatic and cardiometabolic parameters of control individuals as several studies have indicated that initial periodontal treatment has a positive effect on hemostatic and cardiometabolic parameters [30–34].

Additionally, we measured periodontitis using PISA. PISA represents the amount of inflamed periodontal tissue better than any other continuous measurement (e.g., mean probing pocket depth). PISA has inherent statistical advantages (less misclassification and more power) over established periodontitis classifications that report discrete rather than continuous values [19, 35].

Before making conclusions, limitations should be considered. Although we consider the use of the PISA to quantify periodontal burden as a strength of our study, some issues remain. PISA estimates the inflammatory tissue area, but does not capture other quantitative (microbial load) and qualitative (microbe type) parameters which determine systemic inflammation and subsequent risk of another disease [19]. We expect these sources of measurement error would dilute effect sizes found, but the extent is difficult to ascertain [36]. This justifies also analysing the current data per group. The group-wise comparison results are consistent with those from the analysis incorporating PISA. Furthermore, there are some possible issues with potential confounding. The self-reported BMI measurements in the control group may have led to erroneous adjusted estimates. We explored this by imputing validated corrections of the self-reported BMIs, but our overall conclusions from the cross-sectional analysis did not change. A further possible residual confounder could have been the presence of inflammation due to another infection, obscuring the effect of periodontitis. A sensitivity analysis excluding individuals with outlier CRP values was done to explore this, and did not produce different results from the main analysis. Finally, and more specifically the cross-sectional association between von Willebrand factor and PISA could suffer from residual confounding due to imprecision in measurement of smoking- which is known to be correlated with vWF levels. We explored this further by analysing current smoking intensity, but this did not importantly change results. The potential for residual confounding due to measurement error in smoking as well as not finding any association in the longitudinal analysis between von Willebrand factor and PISA leads us to judge that we have not found convincing evidence of a causal relationship between periodontitis and von Willebrand factor.

It is important to stress the population studied. The control individuals were, on average, younger (mean 45 years) than the typical population at risk of cardiovascular disease and VTE (a median age of 66 years) in primary prevention statin trials [37], and a mean age of 57 years in VTE trials [38]. As shown by the aforementioned meta-analysis by Teeuw et al.(2014) [9] and reinforced by a recent analysis by Orlandi et al. (2022) [39], it so far appears that periodontitis interventions only appear to lead to improvements in cardiometabolic parameters in populations who already have established cardiovascular disease. Further evidence of the systemic benefits has accumulated since this meta-analysis including glycaemic control in people with diabetes as well as an improvement in the cardiovascular risk profile of people with metabolic syndrome [40, 41]. With regards to haemostatic biomarker findings, our study is also in agreement with previous analyses. This suggests that periodontitis does not elicit systemic effects on its own; it elicits a disturbed state in concert with other pathophysiological processes, and periodontal intervention in this setting partially restores the disturbed state. Although our study results imply that treating periodontitis may not be a sensible means of primary prevention, it is likely an effective means of secondary prevention in individuals with cardiovascular disease and individuals with diabetes- where questions remain about practical implementation.

With regards to secondary prevention of VTE, we should perhaps stress that our study does not exclude whether periodontitis treatment is an effective means of secondary prevention in individuals with (increased risk of) VTE. Therefore, efforts should be made to investigate whether periodontal interventions improve haemostatic parameters of participants who are at higher risk of VTE. The most obvious candidates would be individuals with VTE who have stopped their anticoagulant treatment, as done by Biederman et al.(2018) [42] by randomizing VTE individuals into statin and non-statin treatment groups. Another approach would be to investigate the effect of periodontal interventions in individuals with a generally higher risk of VTE. Participants with obesity problems would be an interesting field of study, given that the population’s risk of VTE is largely attributable to obesity [43].

In conclusion, this study shows hardly any evidence for a correlation between periodontitis and cardiometabolic and haemostatic parameters nor an effect of periodontal intervention on these parameters in control individuals with periodontitis.

## Electronic supplementary material

Below is the link to the electronic supplementary material.

Supplementary Material 1

## Data Availability

No datasets were generated or analysed during the current study.

## Declarations

Dr. Meijer reports speaker fees from Bayer and Alexion, participating in a trial steering committee for Bayer, and consulting fees from Uniqure. The other authors have nothing to declare.
