# Supplementary material for: Association of periodontitis with cardiometabolic and haemostatic parameters
Source: Clin Oral Investig. 2024 Aug 30;28(9):506. doi: 10.1007/s00784-024-05893-y (PMC11364793; doi:10.1007/s00784-024-05893-y)
Supplement: Supplementary file 1 — Supplementary Material 1 [file 784_2024_5893_MOESM1_ESM.docx]

**Coagulation biomarker assays**

Factor VIII and d-dimer were analysed using ACL300 top; the reagents were used according to the manufacturers’ instructions.

Von Willebrand antigen was analysed with ELISA using antibodies supplied by Dako (Heverlee, Belgium).

The thrombin generation assays (TGA) were done with commercially available reagents from Thrombinscope/Stago (Leiden, the Netherlands), performed in PPP with the fluorimetric method described by Hemker et al. (2002), namely Calibrated Automated Thrombography. Coagulation was activated with commercially available reagents containing recombinant tissue factor (TF, final concentration: 5 pm), phospholipids (final concentration: 4 µM), with and without the presence of soluble thrombomodulin (TM, the concentration was not given by the manufacturer). The reagents were purchased from Thrombinoscope BV, Maastricht, the Netherlands, and the thrombin generation experiments were executed following the protocols provided by Thrombinoscope.

Clot lysis assay was measured as follows: Lysis of a tissue factor–clot, induced by exogenous tissue-type plasminogen activator (t-PA), was studied by monitoring changes in turbidity during clot formation and subsequent lysis as described previously by Meltzer et al. (2008). In short, 50 μl plasma was pipetted into a 96-well microtiter plate. Subsequently, 50 μl of a mixture containing phospholipid vesicles (40% L-α-dioleoylphosphatidylcholine, 20% L-α-dioleoylphosphatidylserine, and 40% L-α-dioleoylphosphatidylethanolamine, final concentration 10 μM), t-PA (final concentration 56 ng/ml), tissue factor (Innovin [Siemens, Healthcare Diagnostics, Marburg, Germany] final dilution 1:1000), and CaCl2 (final concentration 17 mM), diluted in HEPES buffer (25 mM HEPES [N-2-hydroxytethylpiperazine-N'-2-ethanesulfonic acid], 137 mM NaCl, 3.5 mM KCl, 3 mM CaCl2, 0.1% bovine serum albumin, pH 7.4), was added using a multichannel pipette. After thorough mixing, the plate was incubated at 37 °C in a Spectramax 340 kinetic microplate reader (Molecular Devices Corporation), and the optical density at 340 nm was monitored every 20 s, resulting in a clot-lysis turbidity profile. The clot lysis time (CLT) was derived from this clot-lysis profile and defined as the time (minutes) from the midpoint of the clear to maximum turbid transition, representing clot formation, to the midpoint of the maximum turbid to clear transition, representing the lysis of the clot.

**references**

Hemker, H.C., Giesen, P., AlDieri, R., Regnault, V., de Smed, E., Wagenvoord, R., Lecompte, T. & Beguin, S. 2002, "The calibrated automated thrombogram (CAT): a universal routine test for hyper- and hypocoagulability", *Pathophysiology of haemostasis and thrombosis,* vol. 32, no. 5-6, pp. 249-253.

Meltzer, M.E., Lisman, T., Doggen, C.J., de Groot, P.G. & Rosendaal, F.R. 2008, "Synergistic effects of hypofibrinolysis and genetic and acquired risk factors on the risk of a first venous thrombosis", *PLoS medicine,* vol. 5, no. 5, pp. e97.

**Sensitivity analysis: outliers removed**

| **Between group means** | | | |
| --- | --- | --- | --- |
| **Coagulation parameters** | Total tooth extraction | Control group | p |
| Mean Factor VIII % (SD) (values above 200% removed) | 117 (30) | 106 (23) | 0.05 |
| Mean Endogenous Thrombin potential in nM/min (SD) | 1145 (201) | 1133 (168) | 0.76 |
| **Inflammatory/metabolic parameters** |  |  |  |
| Median C-reactive protein (IQR) | 2.2 (0.9-3.9) | 0.9 (0.4-2.1) | <0.01* |
| n missing | 3 |  |  |
| Mean Total cholesterol in mmol/L (SD) | 5.2 (1.0) | 5.1 (1.1) | 0.88 |
| n missing | 1 | 1 |  |
| Mean LDL in mmol/L (SD) | 3.6 (1.0) | 3.4 (1.1) | 0.22 |
| Mean HDL in mmol/L (SD) | 1.3 (0.4) | 1.6 (0.6) | <0.01 |
| Median HOMA2 IR (IQR) | 0.91 (0.68 – 1.37) | 0.89 (0.62 – 1.22) | 0.83 |
| * Wilcoxon signed rank test  transformation LDL = low density lipoprotein; HDL = high density lipoprotein | | | |

| **Longitudinal values of the subjects undergoing total tooth extraction (n=50)** | | | | | |
| --- | --- | --- | --- | --- | --- |
| **Coagulation parameters** | **Before** | **After** | **Change** | **95% CI change** | **p** |
| Mean Factor VIII % (SD) | 119 (35) | 115 (35) | -3 (21) | -9 - 3 | 0.25 |
| Mean von Willebrand factor % (SD) | 136 (54) | 141 (49) | 5 (51) | -6 - 16 | 0.37 |
| Mean Endogenous Thrombin Potential in nM/min (SD) | 1145 (201) | 1081 (249) | -62 (248) | -125 – 1 | 0.05 |
| Median Endogenous Thrombin potential TM in nM/min (IQR) | 344 (182-463) | 398 (209-495) | -2 (-81 - 106) | -- | 0.93* |
| Median D dimer | 252 (166-378) | 268 (143-421) | -1 (121) | -36-34 | 0.95 |
| Median Clot lysis time | 69 (61-76) | 66 (59-77) | -2 (-7 – 4) | -- | 0.20 |
| **Inflammatory/metabolic parameters** | | | | | |
| median C-reactive protein (IQR) | 2.3 (1.2-4.3) | 1.8 (0.9-3.6) | -0.1 (-0.7 – 0.6) | -- | 0.50* |
| n missing | 3 | 4 | 7 |  |  |
| * Wilcoxon signed rank test | | | | | |

| **Regression analyses: outliers removed** | | |
| --- | --- | --- |
| **Laboratory marker** | **Beta coefficient  1000 mm^2^ PISA increment (95% CI)** | p |
| **Cross-sectional analysis*** | | |
| Factor VIII | 2 (-6 – 9) | 0.65 |
| ETP | 17 (-27 – 58) | 0.61 |
| Clot lysis time | -1.75 (-5.6 – 2.1) | 0.37 |
| CRP (logarithm) | 0.01 (-0.42 – 0.44) | 0.98 |
| Total cholesterol | -0.14 (-0.40 – 0.12) | 0.28 |
| LDL | -0.18 (-0.43 – 0.71) | 0.16 |
| HDL | -0.02 (-0.12 – 0.08) | 0.64 |
| HOMA2 IR | 0.07 (-0.04 – 0.19) | 0.17 |
| **Longitudinal analysis** | **Change score beta coefficient  1000 mm^2^ PISA increment (95% CI)** |  |
| Factor VIII | -2 (-9 – 6) | 0.68 |
| Von Willebrand factor | -2 (-15 – 12) | 0.78 |
| ETP | 0 (-81 – 81) | 0.99 |
| ETP TM | -55 (-132 – 22) | 0.16 |
| Dimer | -12 (-56 – 31) | 0.57 |
| Clot lysis time | -1 (-4 – 2) | 0.52 |
| CRP | 0.1 (-0.7 – 0.9) | 0.80 |
| Total cholesterol | 0.0 (-.3 - 0.3) | 0.85 |
| LDL | 0.0 (-0.2 - 0.3) | 0.81 |
| HDL | 0.0 (0.1 - 0.2) | 0.76 |
| HOMA2 IR | -0.01 (-0.18 – 0.16) | 0.89 |
| * adjusted for age, sex, BMI, smoking status and educational status | | |

**Sensitivity analysis 2: CRP outliers excluded**

| **Cross-sectional analyses, between group comparisons** | | | |
| --- | --- | --- | --- |
| **Coagulation parameters** | Total tooth extraction | Control group | p |
| Mean Factor VIII % (SD) | 119 (34) | 109 (32) | 0.14 |
| Mean von Willebrand factor % (SD) | 148 (62) | 105 (40) | <0.01 |
| Mean Endogenous Thrombin potential in nM/min (SD) | 1143 (224) | 1120 (162) | 0.56 |
| Median Endogenous Thromb potential TM in nM/min (IQR) | 344 (182-523) | 414 (333-590) | <0.01* |
| Median D-dimer | 234 (162-353) | 210 (115-415) | 0.28* |
| Median Clot lysis time | 69 (61-79) | 65 (59-71) | 0.17** |
| Median HOMA2 IR | 0.97 (0.72 – 1.40) | 0.87 (0.61 – 1.23) | 0.35* |
| *Wilcoxon signed rank test **t-test after log transformation | | | |

| **Longitudinal values of the subjects undergoing total tooth extraction (n=50)** | | | | | |
| --- | --- | --- | --- | --- | --- |
| **Coagulation parameters** | **Before** | **After** | **Change** | **95% CI change** | **p** |
| Mean Factor VIII % (SD) | 117 (32) | 116 (34) | -1 (26) | -9 – 7 | 0.85 |
| Mean von Willebrand factor % (SD) | 141 (53) | 140 (55) | 0 (51) | -16 – 15 | 0.95 |
| Mean Endogenous Thrombin Potential in nM/min (SD) | 1145 (234) | 1087 (256) | -58 (248) | -134 – 18 | 0.13 |
| Median Endogenous Thrombin potential TM in nM/min (IQR) | 344 (182-463) | 300 (211-555) | -14 (-94 - 87) | -- | 0.57* |
| Median D dimer (IQR) | 260 (164-361) | 266 (140-392) | -2 (158) | -50 – 47 | 0.94 |
| Median Clot lysis time (IQR) | 69 (61-81) | 66 (60-79) | -2 (-8 - 4) | -- | 0.18 |
| **Inflammatory/metabolic parameters** | | | | | |
| median C-reactive protein (IQR) | 2.2 (0.8-4.1) | 1.8 (0.9-4.1) | 0 (-0.8 - 1.2) | -- | 0.86* |
| n missing | 3 | 4 | 7 |  |  |
| Median HOMA2 IR (IQR) | 1.05 (0.76–1.43) | 0.89 (0.60–1.50) | -0.09 (-0.41–0.22) | -- | 0.18* |
| * Wilcoxon signed rank test | | | | | |

| **Regression analyses** | | |
| --- | --- | --- |
| **Laboratory marker** | **Beta coefficient  1000 mm^2^ PISA increment (95% CI)** | p |
| **Cross-sectional analysis*** | | |
| Factor VIII | 3 (-6 – 13) | 0.46 |
| Von Willebrand factor | 27 (12 - 43) | <0.01 |
| ETP | 54 (4 – 104) | 0.03 |
| ETP TM | 30 (-24 – 84) | 0.27 |
| D-dimer (logarithm) | 0.0 (-0.3-0.3) | 0.88 |
| Clot lysis time | -1.6 (-5.8 – 2.5) | 0.42 |
| HOMA2 IR | 0.08 (-0.08 – 0.26) | 0.29 |
| **Longitudinal analysis** | **Change score beta coefficient  1000 mm^2^ PISA increment (95% CI)** |  |
| Factor VIII | -1 (-11 – 9) | 0.87 |
| Von Willebrand factor | 3 (-16 – 22) | 0.76 |
| ETP | -22 (-116 – 72) | 0.63 |
| ETP TM | -5 (-130 – 119) | 0.16 |
| Dimer | -29 (-87 – 29) | 0.32 |
| Clot lysis time | 0 (-4 – 4) | 0.92 |
| HOMA2 IR | 0.04 (-0.25 – 0.33) | 0.78 |

* adjusted for age, sex, BMI, smoking status and educational status

**Sensitivity analysis 3: exclusion of participants with provoking factors/history of VTE**

| **Between group means** | | | |
| --- | --- | --- | --- |
| **Coagulation parameters** | Total tooth extraction (n=54) | Control group (n=50) | p |
| Mean Factor VIII % (SD) | 116 (31) | 113 (33) | 0.69 |
| Mean von Willebrand factor % (SD) | 145 (65) | 106 (43) | <0.01 |
| Mean Endogenous Thrombin potential in nM/min (SD) | 1091 (228) | 1152 (166) | 0.17 |
| Median Endogenous Thromb potential TM in nM/min (IQR) | 393 (188-500) | 417 (332-592) | <0.01* |
| Median D-dimer | 243 (162-353) | 205 (124-439) | 0.23* |
| Median Clot lysis time | 70 (58-75) | 67 (62-76) | 0.86* |
| * Wilcoxon signed rank test | | | |

| **Longitudinal values of the subjects undergoing total tooth extraction (n=50)** | | | | | |
| --- | --- | --- | --- | --- | --- |
| **Coagulation parameters** | **Before** | **After** | **Change** | **95% CI change** | **p** |
| Mean Factor VIII % (SD) | 118 (35) | 116 (36) | -0 (24) | -8 - 7 | 0.96 |
| Mean von Willebrand  factor % (SD) | 139 (62) | 139 (57) | 0 (52) | -17 – 17 | 0.99 |
| Mean Endogenous Thrombin Potential in nM/min (SD) | 1091 (230) | 1042 (219) | -50 (216) | -120 - 21 | 0.30 |
| Median Endogenous Thrombin potential TM in nM/min (IQR) | 316 (167-418) | 289 (213-468) | -1 (-61 - 103) | -- | 0.60* |
| Median D dimer | 260 (164-361) | 266 (140-431) | 6 (141) | -40 - 52 | 0.78 |
| Median Clot lysis time | 70 (58-76) | 66 (57-72) | -2 (-7 - 4) | -- | 0.12 |
| * Wilcoxon signed rank test | | | | | |

| **Regression results** | | |
| --- | --- | --- |
| **Laboratory marker** | **Beta coefficient  1000 mm^2^ PISA increment (95% CI)** | **p** |
| **Cross-sectional analysis*** | | |
| Factor VIII | 5 (-5 – 14) | 0.36 |
| Von Willebrand factor | 19 (1 - 37) | 0.03 |
| ETP | 34 (-22 – 90) | 0.23 |
| ETP TM | 33 (-28 – 94) | 0.28 |
| D-dimer (logarithm) | 0.0 (-0.3-0.3) | 0.88 |
| Clot lysis time | -2 (-6 – 2) | 0.31 |
| **Longitudinal analysis** | **Change score beta coefficient**  **1000 mm^2^ PISA increment (95% CI)** |  |
| Factor VIII | -1 (-11 – 8) | 0.78 |
| Von Willebrand factor | 8 (-12 – 28) | 0.41 |
| ETP | -50 (-133 – 33) | 0.23 |
| ETP TM | -27 (-110 – 55) | 0.51 |
| Dimer | -23 (-78 – 32) | 0.40 |
| Clot lysis time | -1 (-5 – 3) | 0.69 |
| * adjusted for age, sex, BMI, smoking status and educational status | | |

**Sensitivity analysis 4: imputation of BMI correction due to weight underreports**

| **Regression results** | | |
| --- | --- | --- |
| **Laboratory marker** | **Beta coefficient  1000 mm^2^ PISA increment (95% CI)** | **p** |
| **Cross-sectional analysis*** | | |
| Factor VIII | 1 (-7 – 10) | 0.74 |
| Von Willebrand factor | 23 (8 - 38) | <0.01 |
| ETP | 37 (-11 – 84) | 0.13 |
| ETP TM | 20 (-33 – 74) | 0.45 |
| D-dimer (logarithm) | 0.0 (-0.3 – 0.3) | 0.96 |
| Clot lysis time | -2 (-5 – 2) | 0.41 |
| CRP (logarithm) | -0.13 (-0.39 – 0.13) | 0.33 |
| Total cholesterol | -0.19 (-0.48 – 0.09) | 0.17 |
| LDL | -0.23 (-0.50 – 0.05) | 0.10 |
| HDL | 0.01 (-0.12 – 0.13) | 0.93 |
| * adjusted for age, sex, BMI, smoking status and educational status | | |

**Sensitivity analysis 5: regression of longitudinal HDL values for subjects with baseline HDL-C values below optimum HDL concentrations**

| **Longitudinal analysis** | **Change score beta coefficient**  **1000 mm^2^ PISA increment (95% CI)** |  |
| --- | --- | --- |
| HDL, all values | 0.08 (-0.01 – 0.14) | 0.02 |
| HDL, baseline < 1.7 mmol/L | 0.05 (-0.01 – 0.10) | 0.10 |
| HDL, baseline < 1.6 mmol/L | 0.04 (-0.03 – 0.10) | 0.25 |
| HDL, baseline < 1.5 mmol/L | 0.02 (-0.04 – 0.09) | 0.45 |

| **Regression results: cross-sectional analysis** | | | |
| --- | --- | --- | --- |
| **Laboratory marker** | **Beta coefficient  1000 mm^2^ PISA increment (95% CI)** | |  |
|  | | **Cross-sectional analysis*** | |
| Factor VIII | 0 (-9 – 9) | | 0.95 |
| Von Willebrand factor | 21 (6 - 35) | | <0.01 |
| ETP | 28 (-22 – 79) | | 0.26 |
| ETP TM | 19 (-36 – 74) | | 0.45 |
| D-dimer (logarithm) | 0.0 (-0.3 – 0.3) | | 0.77 |
| Clot lysis time | -1 (-5 – 2) | | 0.46 |
| CRP (logarithm) | -0.18 (-0.39 – 0.13) | | 0.24 |
| Total cholesterol | -0.19 (-0.48 – 0.09) | | 0.24 |
| LDL | -0.21 (-0.50 – 0.07) | | 0.14 |
| HDL | 0.01 (-0.12 – 0.13) | | 0.91 |
| HOMA2 IR | 0.03 (0.09-0.16) | | 0.60 |
|  | | * adjusted for age, sex, BMI, smoking status and educational status | |

**Sensitivity analysis 6: exclusion of antibiotic use**

| **Regression results: longitudinal analysis** | | |
| --- | --- | --- |
| **Laboratory marker** | **Change score beta coefficient**  **1000 mm^2^ PISA increment (95% CI)** | **p** |
| **Cross-sectional analysis*** | | |
| Factor VIII | 1 (-9 – 11) | 0.85 |
| Von Willebrand factor | 10 (-8 - 28) | 0.26 |
| ETP | -23 (-113 - 67) | 0.60 |
| ETP TM | -3 (-113 - 66) | 0.95 |
| D-dimer (logarithm) | -20 (-76-36) | 0.47 |
| Clot lysis time | 0 (-4 – 4) | 0.95 |
| CRP | 1 (-.5-3) | 0.15 |
| Total Cholesterol | 0.1 (-0.1-0.4) | 0.29 |
| LDL | 0.2 (-0.1-0.4) | 0.17 |
| HDL | 0.07 (0.01-0.14) | 0.03 |
| HOMA 2 IR | -0.03 (-0.27 – 0.20 | 0.76 |

**Sensitivity analysis 7: smoking intensity as opposed to smoking status**

| **Laboratory marker** | **Coefficient*  1000 mm^2^ PISA increment (95% CI) smoking modelled as smoking intensity** | **Subgroup analysis only smokers, with smoking intensity modelled** |
| --- | --- | --- |
| Factor VIII | 0 (-8 to 8) | 3 (-5-12) |
| Von Willebrand factor | 21 (6 to 35) | 25 (7-43) |
| ETP | 28 (-19 to 75) | 57 (-9 – 125) |
| ETP TM | 25 (-23 to 79) | 3 (-60 – 68) |
| Dimer (logarithm) | -0.1 (-0.2 to 0.4) | 0 (-0.3-0.2 |
| CLT | -2 (-6 to 1) | -1 (-6-4) |
| CRP (logarithm) | -0.18 (-0.47 to 0.12) | -0.14 (-.52 - .24) |
| Total cholesterol | -0.2 (-0.4 to 0.1) | -0.22 (-0.53-0.10) |
| LDL | -0.2 (-0.4 to 0.1) | -0.20 (-.5 - .1) |
| HDL | 0.01 (-0.11 to 0.12) | -0.02 (-0.15-0.09) |

**Scatter plots depicting the relationship between PISA and haemostatic profiles**

*Cross-sectional data*


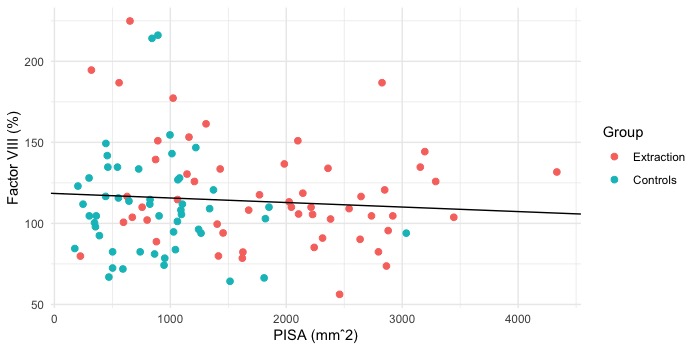

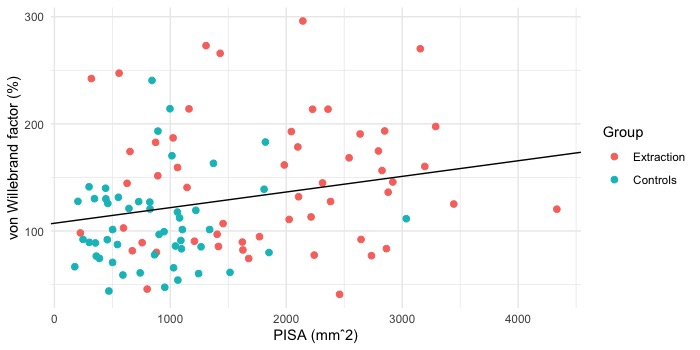


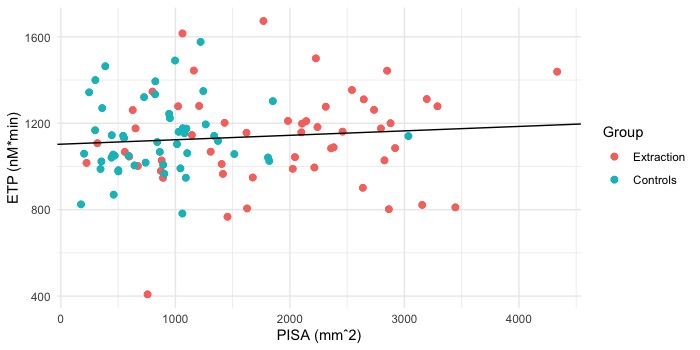


**
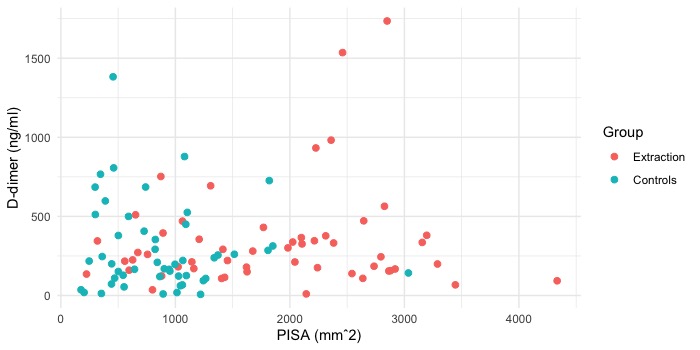
**

**
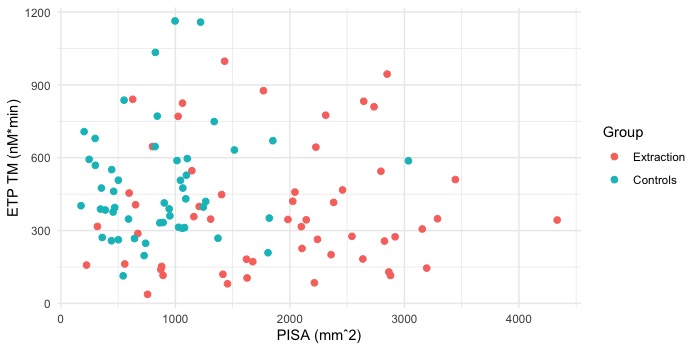
**

**
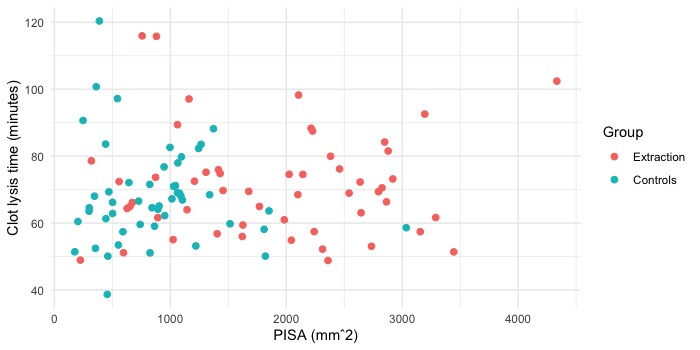
**

*Longitudinal data*


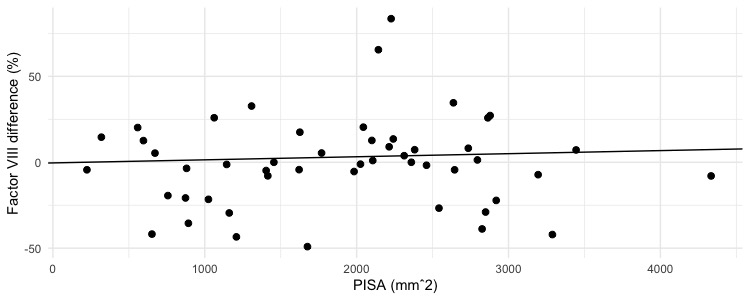


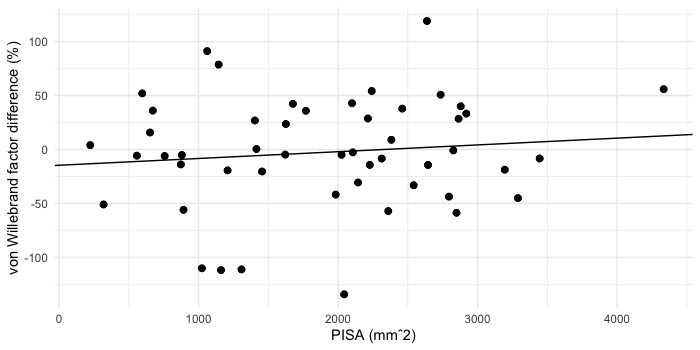


**
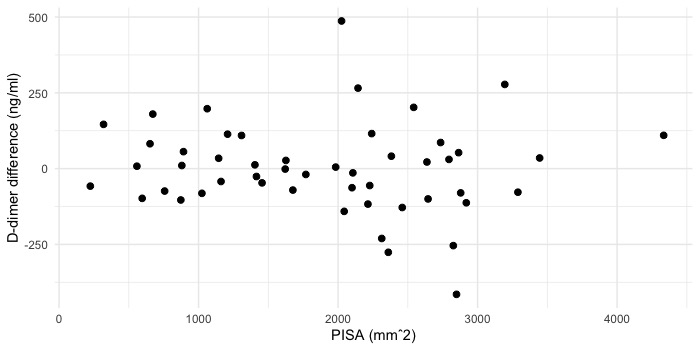
**

**
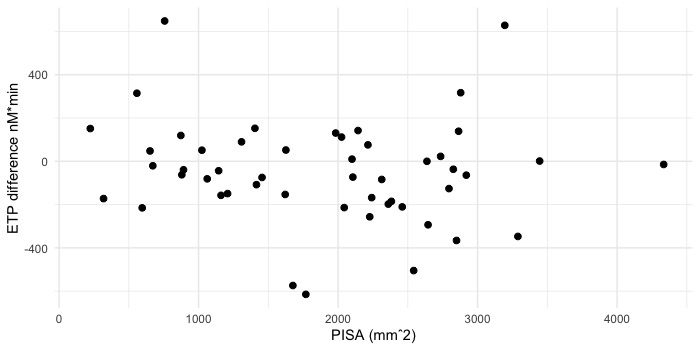
**
